# S100 Calcium Binding Protein A16 Promotes Cell Proliferation by triggering LATS1 ubiquitin degradation mediated by CUL4A ligase to inhibit Hippo pathway in Glioma development

**DOI:** 10.7150/ijbs.79924

**Published:** 2023-04-02

**Authors:** Yifang Hu, Rihua Zhang, Shan Lu, Wensong Zhang, Dan Wang, Yaoqi Ge, Feng Jiang, Xiaoxuan Qin, Yun Liu

**Affiliations:** 1 Department of Geriatric Endocrinology, The First Affiliated Hospital of Nanjing Medical University, Nanjing, Jiangsu, China; 2 Department of Core Facility Center, The First Affiliated Hospital of Nanjing Medical University, Nanjing, Jiangsu, China; 3 Department of Pharmacy, The First Affiliated Hospital of Nanjing Medical University, Nanjing, Jiangsu, China; 4 Department of Neonatology, Obstetrics and Gynecology Hospital of Fudan University, Shanghai, China; 5 Department of Neurology, The First Affiliated Hospital of Nanjing Medical University, Nanjing, China

**Keywords:** glioma, S100A16, tumor progression, Hippo pathway, LATS1

## Abstract

**Background:** S100 Calcium Binding Protein A16 (S100A16), a novel member of S100 protein family, is linked to tumorigenic processes and abundantly expressed in CNS tissues. Our study aimed to explore the biological function and possible mechanism of S100A16 in the progression of glioma.

**Methods:** Sequence data of S100A16 and survival prognosis of glioma patients were initially analyzed using public databases. Glioma tissues were collected to examine S100A16 expression levels. Glioma cell lines and nude mice were subjected to *in vitro* and *in vivo* functional experiments. Western blot, immunofluorescence (IF), immunoprecipitation (IP) and ubiquitination assays were done to further elucidate the underlying mechanism.

**Results:** This study firstly revealed that S100A16 was markedly up-regulated in glioma, and patients with higher S100A16 levels have a shorter survival time. S100A16 overexpression promoted the proliferation, invasion and migration of glioma cells, and the tumor formation of nude mice. Importantly, we identified S100A16 as a negative regulator of the Hippo pathway which could decrease LATS1 expression levels, promote the YAP nuclear import and initiate the downstream target genes CYR61 and CTGF. Moreover, our data showed that S100A16 destabilized LATS1 protein by inducing the CUL4A-mediated LATS1 ubiquitination degradation.

**Conclusions:** This study demonstrated a vital biological role of S100A16 in glioma progression mechanism by promoting CUL4A-mediated LATS1 ubiquitination to inhibit Hippo signaling pathway. S100A16 could be a novel biomarker and treatment option for glioma patients.

## Introduction

Glioma is a primary intracranial tumor that originates from glial cells, which account for approximately 24.5% of all brain tumors, as well as 80.9% of malignant tumors 1. Based on 2021 World Health Organization (WHO) criteria, glioma is divided into four grades (1-4), with WHO grade 1 and grade 2 being low-grade glioma (LGG) and WHO grade 3 and grade 4 being high-grade glioma (HGG)^2^. Glioblastoma (GBM), as the WHO grade 4 glioma, is characterized by high aggressiveness, recurrence, disability and mortality rates, with a median 5-year survival rate of only about 15 months 3. Despite advances in surgical resection, and post-operative temozolomide (TMZ) chemotherapy and radiotherapy, there has been limited improvement in patient survival time 4,5. The blood-brain barrier of central nervous system (CNS), which is constituted of endothelial cells, capillaries and basement membrane, prevents most anti-tumor medicines from reaching the brain, posing a severe hurdle for drug research 6. Therefore, new biomarkers and molecular targets for glioma patients are urgently required to improve their prognosis.

The S100 family is a class of small acidic calcium-binding proteins that act as calcium sensors or extracellular factors to regulate a variety of biological processes. S100 proteins are involved in numerous tumor behavior, such as cell proliferation, invasion, angiogenesis and immune escape 7,8. To date, 25 known members of S100 family have been discovered, which have similar molecular structures and exist as dimers or multimers. Each monomer consists of two helix-loop-helix (EF-hand) motifs, a classical C- terminal portion, and an S100-specific N-terminal portion 9. S100 Calcium Binding Protein A16 (S100A16), is a novel S100 family member with a specific structure that lacks N-terminal glutamate residues, resulting in a more hydrophobic effect and a more stable structure than other members. S100A16 is found to be widely expressed in various tissues and is associated with inflammation, adipogenesis, and glucose metabolism 10-12. However, the function of S100A16 in carcinogenesis is complicated, its specific mechanism has not been elucidated. Previous studies reported that S100A16 was implicated in the development of several cancers, including bladder, gastric, colon and pancreatic cancers 13-16. Considering that S100A16 is abundantly expressed in the nervous system, and glioma is the most frequent CNS cancer, it is essential to elucidate the potential function and mechanism of S100A16 in glioma.

The Hippo signaling pathway, initially discovered in Drosophila, is a highly conserved growth control pathway regulating tissue development and regeneration, as well as cell proliferation and apoptosis 17. Physiologically, the Hippo signaling pathway possesses a tumor-suppressive function. When the Hippo pathway is activated, Tao kinase 1 (Tao-1) phosphorylates MST1/2, and MST/SAV1 helps to activate and promote LATS1/2 phosphorylation, which further phosphorylates YAP/TAZ, leading it to be accumulated in the cytoplasm or ubiquitinated for degradation, thereby limiting its entry into the nucleus to exert biological effects. Meanwhile, MST1/2 can mediate MOB1 phosphorylation, and increase its ability to bind and activate LATS1/2 18. When the Hippo pathway is inactivated, YAP/TAZ enters the nucleus and binds to the transcriptional enhancer TEAD, which regulates the expression of target genes like cyclin E, Axl, Cyr61, and CTGF. YAP is an important transcriptional activator that regulates signal transduction, cell growth and tumorigenesis. Numerous studies have demonstrated that the Hippo pathway could negatively regulate glioma growth, invasion, metastasis, epithelial-to-mesenchymal transition (EMT), cell stemness and TMZ resistance 19,20.

In this study, we aimed to gain a better understanding of S100A16's biological function and molecular mechanism in glioma progression by using bioinformatics analysis, *in vitro* and *in vivo* functional tests. The results indicated that S100A16 predicted poor prognosis in glioma by promoting cell proliferation, migration and invasive abilities via inhibiting LATS1 expression in the Hippo pathway. Our study established a critical role of S100A16 in glioma development, and offered a new direction for glioma diagnosis, therapy and prognosis.

## Materials and Methods

### Bioinformatics analysis

Bioinformatics analyses were carried out using gene expression profiles and survival information from public databases, including GEPIA (http://gepia.cancer-pku.cn/detail.php), CGGA (http://www.cgga.org.cn/), TCGA (https://portal.gdc.cancer.gov/) and GEO database (http://www.ncbi.nlm.nih.gov/geo).

GBM RNA-seq data from TCGA and CGGA databases were used to identify differentially expressed genes (DEGs) between high- and low-S100A16 expression groups. R package “DESeq2” was used to distinguish DEGs, which were presented with heatmaps via R package “heatmap” and volcano maps via R package “ggplot”. *P* < 0.05, log FC > 1.0 were regarded as significantly up-regulated DEGs, whereas *P* < 0.05, log FC < -1.0 were regarded as down-regulated DEGs. KEGG and GO analyses were undertaken to identify DEGs enriched pathways and biological functions.

### Tissue samples

Glioma patient tissues and clinical characteristics were collected from the First Affiliated Hospital of Nanjing Medical University. Normal brain tissues (NBTs) were taken from patients receiving decompressive craniotomy for traumatic brain injury (TBI) as the control group. The post-surgical specimens were confirmed with histopathologic diagnosis based on WHO criteria. All patients gave their written informed consent before the procedure.

### Cell culture and Transfection

The glioma cell lines U87, U251, T98G, LN229, A172, SW1738, N3 were purchased from the Chinese Academy of Sciences and grown in DMEM with 10% FBS and 0.5% Penicillin/Streptomycin. Human astrocyte cell NHA was obtained from University of Bergen, and cultured following the manufacturer's instruction. Cells were propagated in a humidified chamber at 37°C with 5% CO_2_. Logarithmic-phase cells with superior performance were chosen for the present study.

Plasmids sh-S100A16, sh-NC, sh-LATS1, si-CUL4A and S100A16 cDNA, LATS1 cDNA, CUL4A cDNA were transfected into glioma cells by utilizing Lipofectamine® 3000 transfection reagent (Invitrogen, USA). Lentivirus was constructed by Genechem (Shanghai, China), and used to infect cells following the manufacturer's guidelines. After 48h of infection, we cultured cells in DMEM with 1ug/ml puromycin (MCE, China) to obtain stable clones. The target sequences applied in this study were presented in **Table S1**.

### qRT-PCR assay

Total RNA was isolated from cells and tissues utilizing Trizol reagent (Invitrogen, America), then reverse-transcribed to cDNAs utilizing HiScript Synthesis kit (Vazyme, China) according to the manual. PCR mixtures were prepared with SYBR ® Green Master Mix (Vazyme, China), RNA primer, cDNA and ddH_2_O. Real‐time quantitative PCR (qRT-PCR) was finally performed on the StepOnePlus Real-Time PCR system (Applied Biosystems, USA) according to pre-set amplification parameters. The qRT-PCR primers were listed in **Table S2**. The relative expression value of each gene was computed with the 2^-ΔΔCT^ method.

### Western blot assay

Total protein was lysed from cells and tissues utilizing RIPA buffer, and its concentrations were measured using a BCA kit (Beyotime, China). Equal amounts of protein were separated by SDS-PAGE, then electro-transferred onto nitrocellulose (NC) membrane (Pall, USA). The membrane was blocked with 5% skimmed milk for 2h at room temperature (RT). Blots were incubated with primary antibodies overnight at 4℃, washed with 1×TBST (3×10 min), and subsequently incubated with secondary antibodies for 2h at RT. Protein bands were exposed with an enhanced chemiluminescence reagent (Beyotime, China), and visualized by gel analysis imaging system (Tanon, China). The following antibodies were used: S100A16, pLATS1 and pMST1/2 (Proteintech, China), β-Actin (Bioss, China), LATS1, MST1, MST2, SAV, pMOB, MOB, pYAP, YAP/TAZ, Ub, CUL4A (Cell Signaling Technology, USA), and DCAF1, DDB1 (Abcam, UK).

### Immunoprecipitation (IP) assay

After cell lysis, equal amounts of protein were taken to incubate with primary antibody (Cell Signaling Technology, USA) or IgG (Beyotime, China) overnight at 4°C. Then, the protein-antibody immunoprecipitates were collected by protein A/G magnetic beads (MCE, China) for 2h at RT. Finally, the complexes were washed with PBS, boiled in loading buffer, and subjected to western blot. For the ubiquitination assay, cells were pre-treated with 10μM MG132 for 8h before IP analysis.

### Nuclear and cytoplasmatic fractionation

To identify YAP intracellular distribution, nuclear and cytoplasmic sections were isolated with Nuclear and Cytoplasmic Extraction Reagents consisting of CER I, CER II and NER (Thermo Fisher Scientific, China), following the manufacturer's guidelines. Histone H3 (Cell Signaling Technology, USA) and β-Tubulin (Proteintech, China) were used as loading controls for cytoplasmic and nuclear proteins, respectively.

### Immunohistochemistry (IHC) assay

Tissues were fixed in 4% paraformaldehyde and embedded in paraffin, then cut into 5 um slices for IHC. Paraffin slices were deparaffinized in xylene and hydrated in gradient alcohol. After antigen repair, tissues were immersed in 3% H_2_O_2_ for 10 min to prevent endogenous peroxidase activity. The slides were incubated with 5% goat serum for 30 min at RT, then incubated overnight at 4°C with S100A16 (Proteintech, China), LATS1 antibody (Cell Signaling Technology, USA), or stained with H&E. After being washed with PBS, tissues were added with secondary antibody binding to primary antibody for 1 h at RT. Streptavidin-biotin complex (SABC) and Diaminobenzidine (DAB) were used to visualize the immunostaining. Finally, sections were counterstained with hematoxylin, dehydrated with alcohol, dried and covered in resin. The stained samples were reviewed and analyzed under a microscope (Nikon, Japan).

### Immunofluorescence (IF) assay

Tissue fixation, deparaffinization, hydration, and antigen repair were the same as the IHC procedure. Cells were fixed with 4% paraformaldehyde for 30 min. Next, tissues or cells were permeabilized with 0.1% Triton-X 100, and blocked with 5% BSA for 30 min. Then, they were incubated overnight with primary antibodies. A TRITC-labelled goat anti-Rabbit IgG (KeyGen Biotech, China) was used as secondary antibody for 1 h at RT in the dark. DAPI was employed to stain the nuclei for another 10 min. IF images were observed under a light microscope (Olympus, Japan) or Confocal microscope (Zeiss, Germany).

### Cell counting kit-8 (CCK-8) assay

GBM cells were seeded in 96-well plates (2,000 cells/well) in triplicate overnight. Next, the cell growth was assayed at different time points with a CCK-8 kit (Beyotime, China) following the manufacturer's protocol. Absorbance at 450 nm was measured by a microplate reader (Thermo, USA).

### Colony formation assay

GBM cells were plated in 6-well plates (500 cells / well) and cultured in a 37 °C 5% CO_2_ incubator for 2 weeks. Colonies were subsequently fixed and stained with 4% paraformaldehyde and Crystal violet. The colony-forming capacity was compared by counting the number of colonies.

### EdU assay

GBM cells were seeded in 96‐well plates (1×10^5^ cells/well) in triplicate overnight. Cells were stained with 50 μM EdU (Ribobio, China) for 2 h, fixed with 4% paraformaldehyde and permeabilizated with 0.5% Triton X‐100. After three PBS washes, they were incubated with 100 µL of 1 × Apollo® reaction for 30 min, followed by 1×Hoechst33342 staining for another 30 min. The stained cells were photographed by fluorescence microscopy (Olympus, Japan).

### Transwell invasion test

GBM cells were added into the top of 24-well Transwell chambers (1×10^5^ cells/well) pre-coated with Matrigel (BD Biosciences, USA) in serum-free DMEM. The bottom chambers were filled with DMEM containing 10% FBS. After incubation for 24 h, the penetrated cells were fixed with 4% methanol, then stained with 0.1% crystal violet. Each well was randomly photographed and counted by a light microscope (Olympus, Japan).

### Wound‐healing test

GBM cells were plated in 6-well plates and cultured to 70-80% confluence. Next, the scratches on the cell were created by a sterile pipette tip. At 0h, 24h or 48h after scratch, the linear wound was imaged using a light microscope (Olympus, Japan). The scratch healing ratio was determined using the formula: (0h scratch width - 48h scratch width) / 0h scratch width × 100%.

### Animal studies

To establish a subcutaneous xenograft model, stable-transfected glioma cells (2×10^6^cells / 100μL) were implanted subcutaneously into the right shoulder of nude mice. Tumor volume was monitored with a vernier caliper weekly. At 30 days after inoculation, xenografts were excised, weighed and photographed. For the intracranial orthotopic model, luciferase-expressing U87 cells (1×10^6^ cells / 10 μL) were injected into the frontal lobes of nude mice brains under a stereotaxic apparatus. Tumor growth was visualized weekly by a bioluminescence imaging system. Mice were monitored for clinical symptoms, moribund animals were euthanized, and brains were removed for the next experiments. Animal procedures were approved by the Institutional Animal Care and Use Committee (IACUC) of Nanjing Medical University (Number: IACUC-2106046).

### Data Analysis

All experiments were done in triplicates and repeated at least three independent times. Statistical analysis was performed in R software 4.0.3, SPSS 14.0 or GraphPad Prism software 5.0. Data were given as mean ± SD. Student t-test was employed to compare two groups, or ANOVA for multiple groups. Kaplan-Meier was utilized to evaluate the survival time. Value of p* < 0.05, p**< 0.01, and p***< 0.001 were defined as statistical significance.

## Results

### S100A16 expression and Survival analyses from Public Database

**Figure 1:**
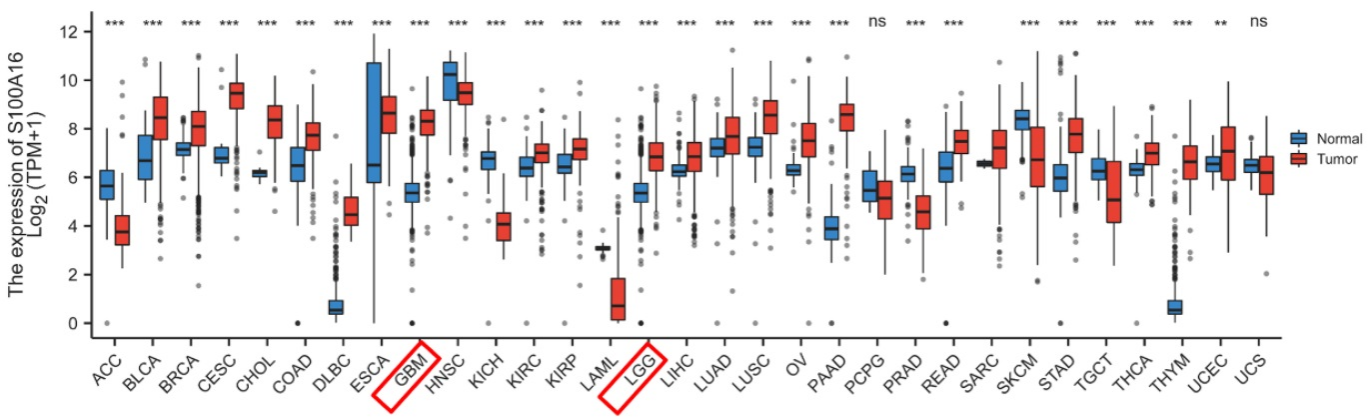
**GEPIA analysis of S100A16 expression status in various tumors.** *p < 0.05, **p < 0.01, ***p < 0.001.

**Figure 2:**
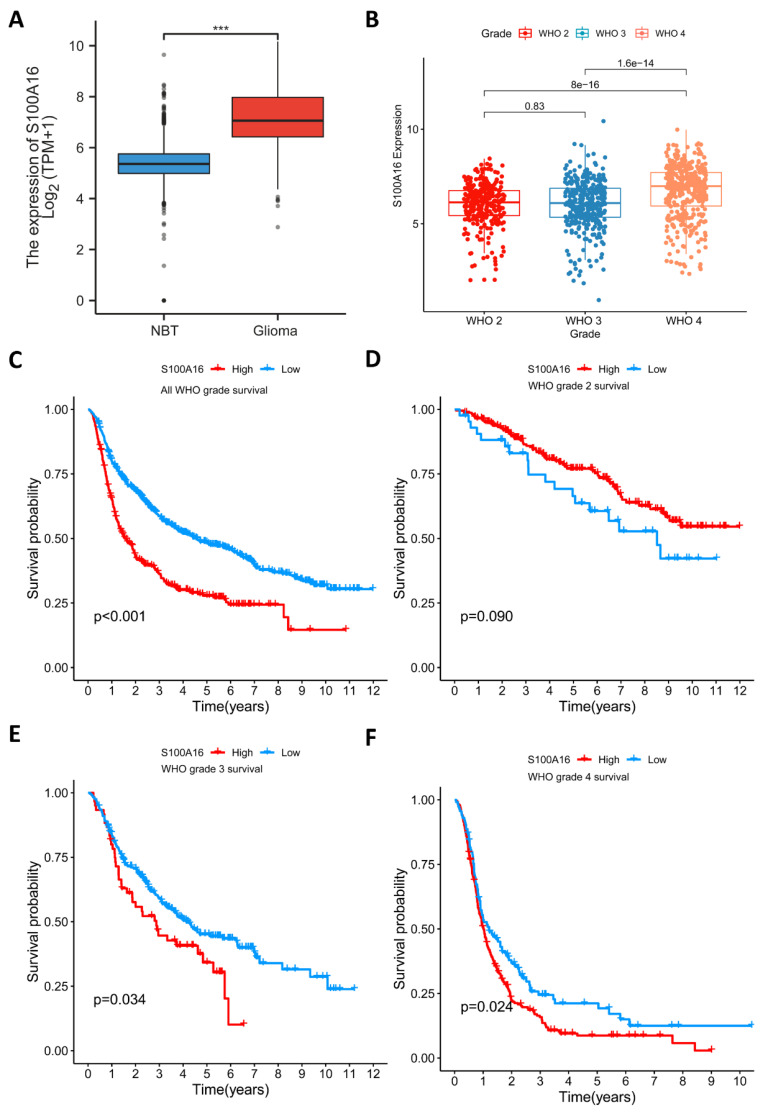
** S100A16 expression levels in glioma and relationship with overall survival. (A)** Comparison of S100A16 expression levels between S100A16 in NBT and GBM from TCGA database. (**B**) S100A16 mRNA expression in different grades of glioma from CGGA database; (**C-F**) Survival analysis of all WHO grade, WHO grade 2, WHO grade 3, and WHO grade 4 glioma (ns: no significant, ***p < 0.001).

S100A16 expression in tumor was first screened in GEPIA database (**Fig. 1**). The result suggested that S100A16 was up-regulated in GBM and LGG, as well as bladder urothelial carcinoma (BLCA), cervical squamous cell carcinoma (CESC), colon adenocarcinoma (COAD) et.al, whereas down-regulated in adrenocortical carcinoma (ACC), esophageal carcinoma (ESCA), and kidney Chromophobe (KICH) et.al. The overexpression of S100A16 in GBM was further examined in TCGA database (**Fig. 2A**). Furthermore, we analyzed S100A16 expression and clinical information of 325 glioma patients based on the CGGA database and showed that the level of S100A16 expression increased with the grade of malignancy, with the highest expression in GBM patients (**Fig. 2B**). Survival analysis showed that high S100A16 expression significantly shortened the survival time of glioma patients, especially for GBM patients (**Fig. 2C-F**). and survival differences were also validated in GEO database (**Fig. S1**).

### Pathway enrichment analyses

**Figure 3:**
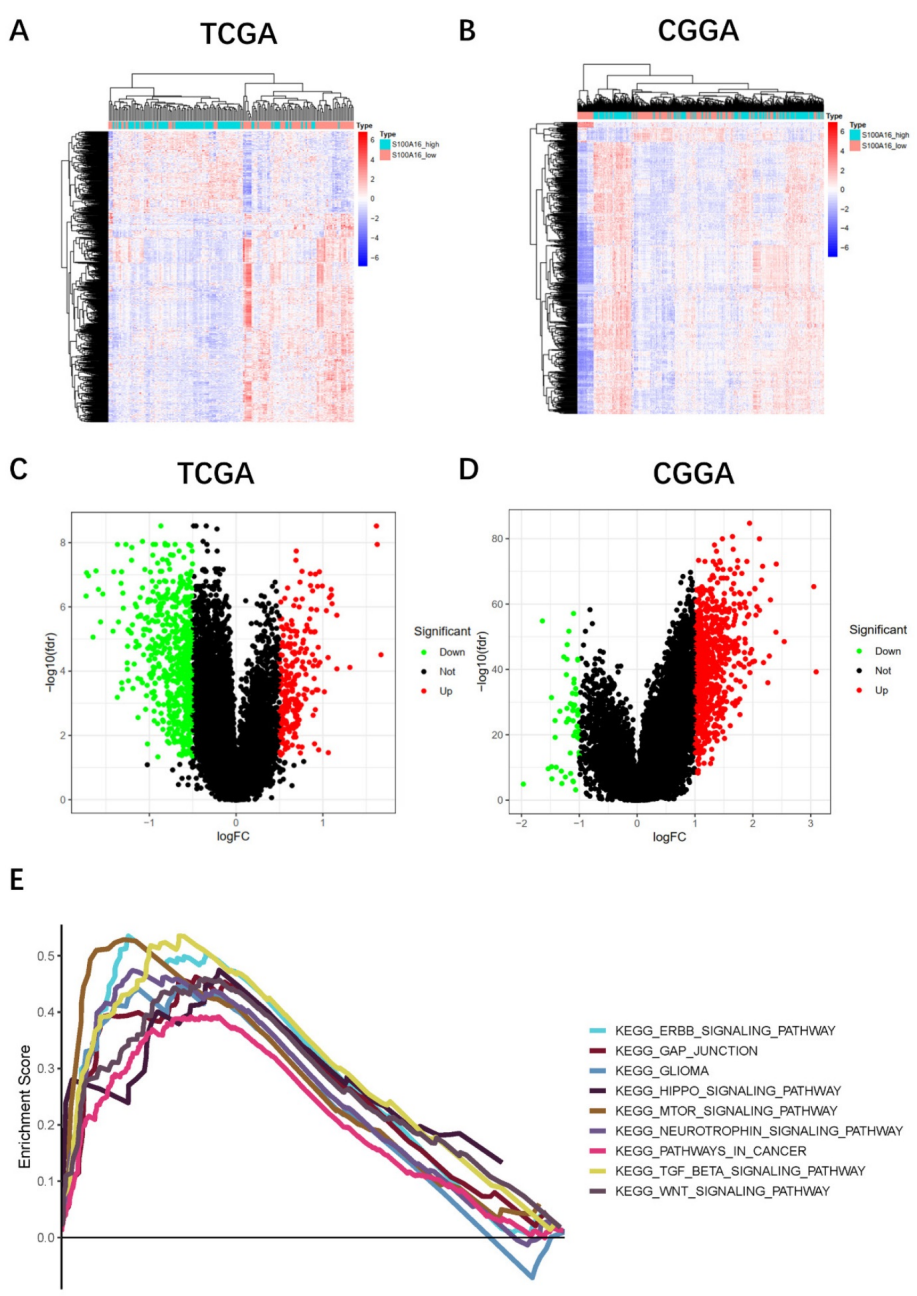
** Pathway enrichment analysis of DEGs between high- and low-S100A16 groups in glioma.** (**A-B**) Heat map of DEGs in TCGA and CGGA database. **(C-D)** Volcano map of DEGs in both databases. **(E)** KEGG enrichment analysis of DEGs (*p* < 0.05).

**Figure 4:**
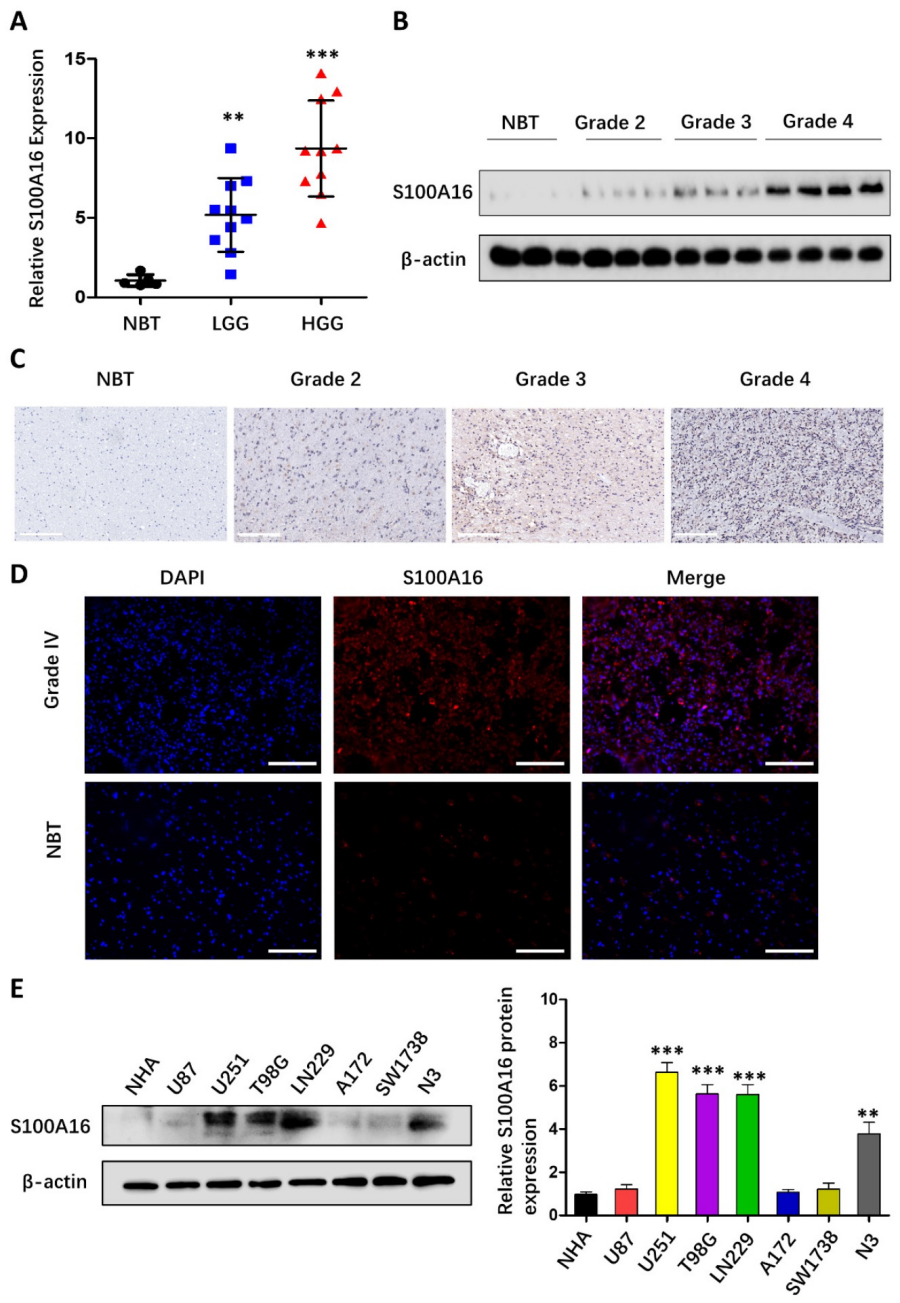
**S100A16 was overexpressed in glioma tissues and cell lines**. **(A)** qRT-PCR analysis of S100A16 mRNA levels in NBTs and different grades of glioma; **(B)** Western Blot analysis of S100A16 protein levels in NBTs and different grades of glioma; **(C)** Representative IHC images of S100A16 in NBTs and WHO II-IV glioma; **(D)** Representative IF images of S100A16 in NBTs and WHO-IV glioma. **(E)** Western Blot analysis of S100A16 protein levels in NHA and different glioma cells. *p < 0.05, **p < 0.01, ***p < 0.001.

To further explore the biological functions and possible pathways that S100A16 enriched, we conducted KEGG and GO pathway enrichment analyses. GBM patients from TCGA and CGGA cohorts were separated into a high-S100A16 group and a low-S100A16 group based on the median value. A total of 879 differential expression genes (DEGs) (|logFC| > 1, FDR < 0.01) were screened and displayed on the hot map (Fig. 3A-B) and volcano map (Fig. 4C-D). Red color denotes up-regulated genes in GBM samples, blue denotes down-regulated genes. GO result suggested that these DEGs were mainly enriched in glial cell development and differentiation, gliogenesis, nervous system myelination, extracellular matrix binding, and calcium-dependent protein binding (Fig. S2). KEGG analysis showed that the DEGs are mainly enriched in the Hippo signaling pathway, mTOR pathway, WNT pathway, CANCER pathway, ERBB pathway, and GLIOMA pathway (Fig. 4E). Among them, the Hippo signaling pathway is the most significantly enriched pathway with a P-value < 0.001 (Fig. S3). Correlation analyses showed that S100A16 was obviously associated with YAP and its target genes CTGF and CYR61 (Fig. S4). These suggested that S100A16 is closely related to the Hippo pathway and cell proliferation.

### S100A16 up-regulation in glioma tissues and cells

To validate the bioinformatic data, we analyzed S100A16 expression in clinical samples from 5 NBTs and 20 glioma tissues of different grades. As seen in **Fig. 4A**, qRT-PCR indicated that S100A16 mRNA levels were significantly up-regulated in HGG (WHOgrade 3-4) and LGG (WHO grade 1-2) compared to NBT. In addition, western blot suggested that S100A16 protein expression was evaluated with increasing glioma grade (**Fig. 4B**). IHC assay also confirmed S100A16 overexpression in glioma, and its positivity was observed in the cytoplasm and nuclei of glioma cells (**Fig. 4C**). IF staining revealed the strong cytosolic expression of S100A16 in GBM relative to NBT (**Fig. 4D**). Next, we analyzed S100A16 expression in glioma U87, U251, T98G, LN229, A172, SW1738, N3 cells and control NHA cells by western blot, and showed different levels of S100A16 proteins (**Fig. 4E**). U251 and T98G cells, presenting the relatively higher expression of S100A16, were chosen for sh-S100A16, while U87 and A172 cells for S100A16 cDNA transfection. These findings revealed that S100A16 was up-regulated in both glioma tissues and cells, which may play a vital role in tumorigenesis and malignancy.

### S100A16 promotes glioma growth, migration and invasion

**Figure 5:**
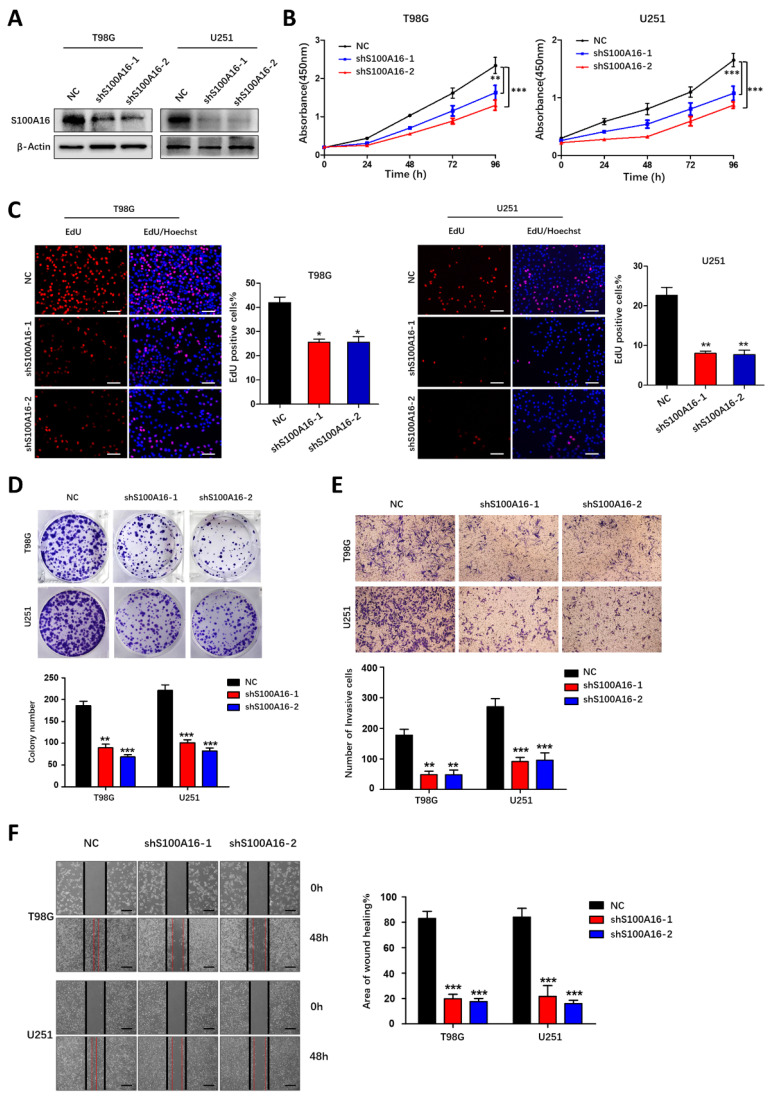
** S100A16 knockdown suppressed proliferation, migration and invasion of glioma cells**.** (A)** Western blot analysis of knockdown efficacy of S100A16 shRNA transfected into T98G and U251 cells. **(B)** CCK-8 assay of cell activity of T98 and U251 infected with sh-S100A16; **(C)** EdU assay of proliferative ability of both infected cells;** (D)** Plate colony assay of proliferative capacity of both infected cells. **(E)** Transwell assay of invasive ability of both infected cells; **(F)** Wound healing test of migration ability of both infected cells. *p < 0.05, **p < 0.01, ***p < 0.001.

**Figure 6:**
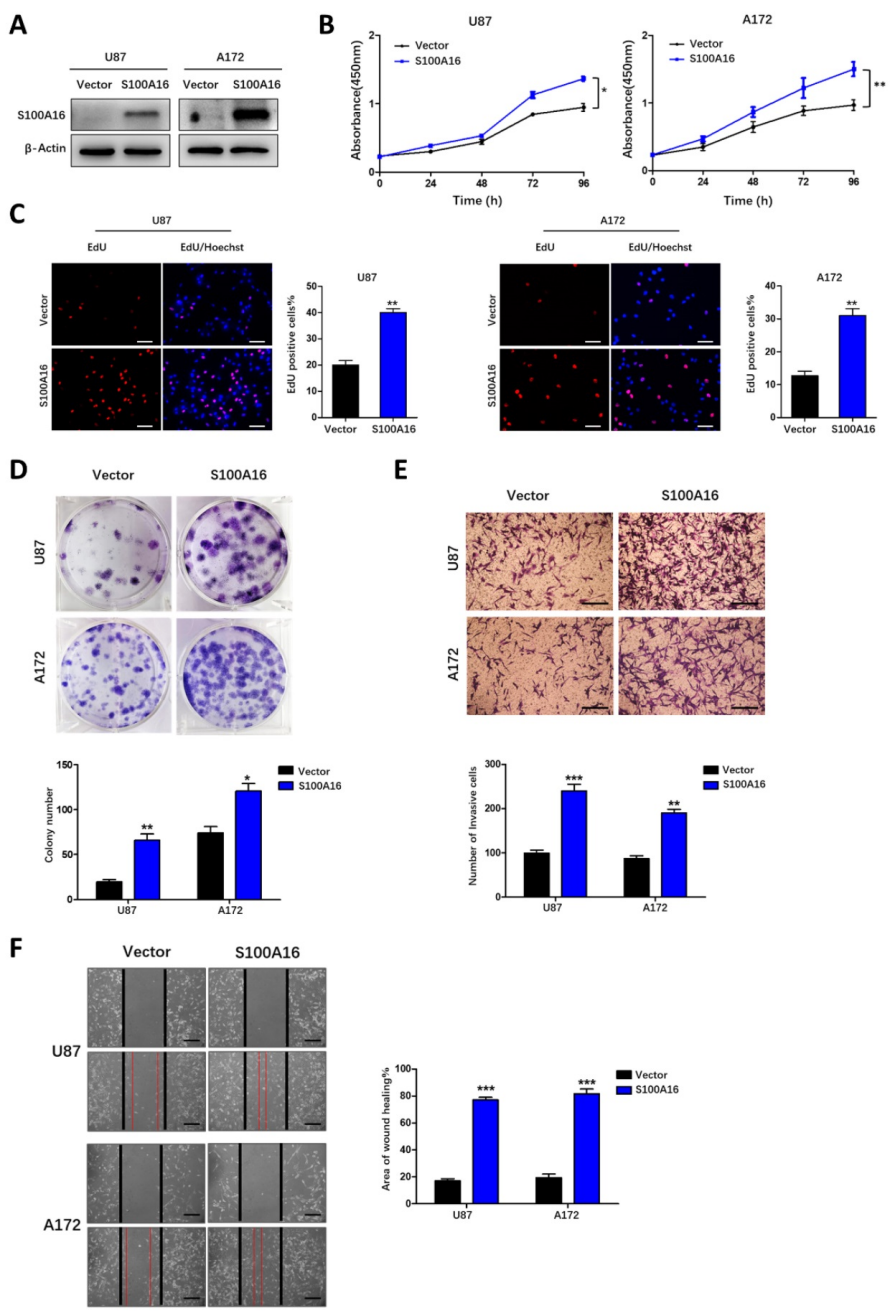
**S100A16 overexpression promoted the proliferation, migration and invasion of glioma cells**.** (A)** Western blot assay of overexpression efficiency of S100A16 cDNA in U87 and A172 cells. **(B)** CCK-8 analysis of cell viability of U87 and A172 transfected with overexpression plasma; **(C)** EdU assay of the proliferative ability in U87- and A172-S100A16 cells;** (D)** Plate colony assay of the proliferative ability in U87- and A172-S100A16 cells. **(E)** Transwell test of invasive capability in U87- and A172-S100A16 cells; **(F)** Wound healing assay of migration ability in U87- and A172-S100A16 cells.

We further conducted functional experiments to investigate the significance of S100A16 in glioma progression. We first confirmed the knockdown efficiency of two sh-S100A16 knockdown in both T98G and U251 cells (**Fig. 5A**), and S100A16 overexpression in both U87 and A172 cells by using western blot (**Fig. 6A**). CCK-8 growth curves showed that silencing S100A16 markedly reduced T98G and U251 cells' growth (**Fig. 5B**), while over-expressing S100A16 increased U87 and A172 cells' growth (**Fig. 6B**). The findings were corroborated in EdU and cloning assays. The EdU-positive cells or colonies were decreased by sh-S100A16 in T98G and U251 cells (**Fig. 5C-D**), but increased by S100A16 overexpressing in U87 and A172 cells (**Fig. 6C-D**). Moreover, we also performed a transwell assay and a wound-healing assay, the results demonstrated that silencing S100A16 by shRNA markedly decreased T98G and U251 invasion cells (**Fig. 5E**) and scratch healing rate** (Fig. 5F)**, and up-regulating S100A16 exhibited elevated A172 and U87 invasion cells (**Fig. 6E**) and healing rate (**Fig. 6F**). Taken together, these findings indicated that S100A16 promoted glioma cells' proliferation, migration, and invasion.

### S100A16 regulates Hippo signaling pathway

**Figure 7:**
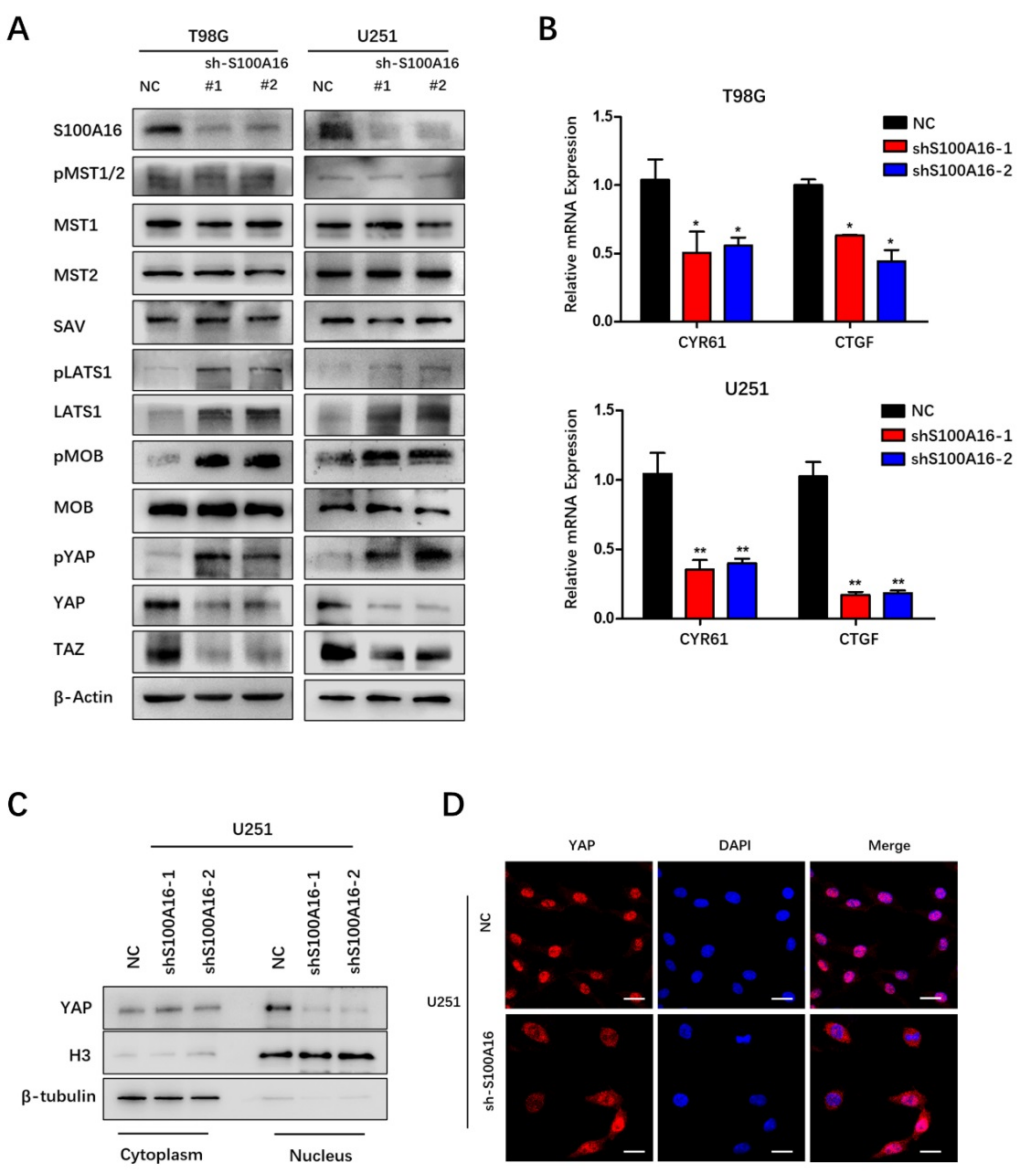
** S100A16 knockdown activates the Hippo signaling pathway. (A)** Western Blot assay of Hippo pathway components' expression in T98G and U251 cells modified with sh-S100A16; **(B)** qRT-PCR of downstream genes CYR61 and CTGF expression levels in modified T98G and U251 cells; **(C)** Western Blot of cytoplasmic and nuclear YAP protein separated from modified U251 cells; **(D)** Immunofluorescence staining of cellular localization of YAP in modified U251 cells.

**Figure 8:**
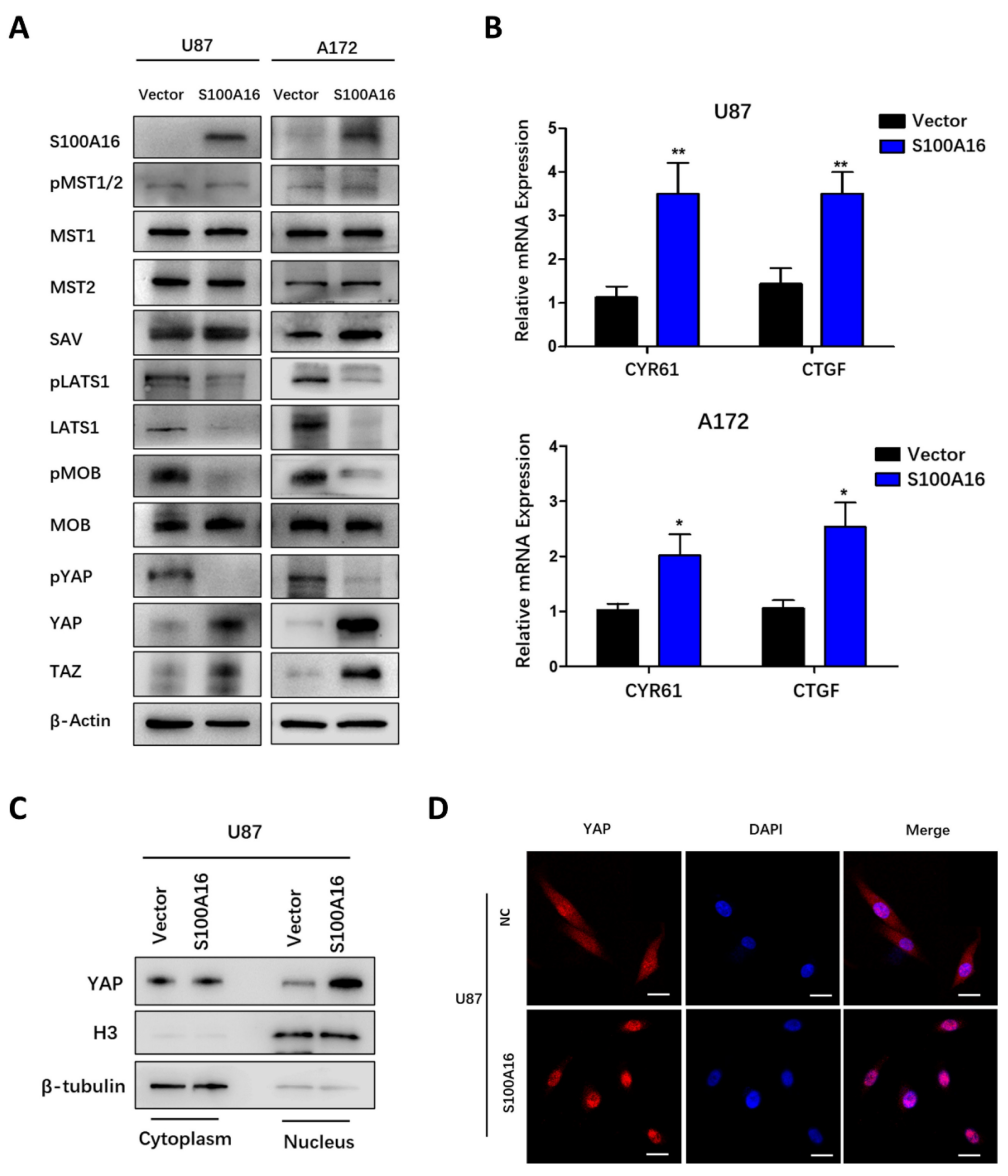
** S100A16 overexpression suppresses the Hippo signaling pathway. (A)** Western Blot assay of Hippo pathway components' expression in U87 and A172 cells modified with over-expressed S100A16 plasma; **(B)** qRT-PCR of downstream genes CYR61 and CTGF in modified U87 and A172 cells; **(C)** Western Blot of cytoplasmic and nuclear YAP protein separated from modified U87 cells; **(D)** Immunofluorescence staining of cellular localization of YAP in modified U87 cells.

Hippo signaling pathway has been previously reported to play a vital role in tumor initiation and progression. Bioinformatics in our study predicted that S100A16 may regulate Hippo signaling pathway in glioma. Therefore, we examined the impact of S100A16 on this pathway's core members in glioma cells by Western blot. As shown in **Fig. 7A**, S100A16-knockdown by shRNA did not change MST1, MST2, pMST1/2 and SAV proteins, but increased LATS1 protein levels and its phosphorylation, and induced MOB phosphorylation while total protein remained constant, accompanied by an increase in YAP phosphorylation while a decrease in total YAP/TAZ protein levels in T98G and U251 cells. In contrast, after overexpression of S100A16, relatively low levels of LATS1, pLATS1, pMOB and pYAP were found in U87 and A172 cells, while total YAP/TAZ proteins were markedly increased (**Fig. 8A**). As the downstream transcriptional regulators of YAP/TAZ, CTGF and CYR61, we used qRT-PCR to examine their mRNA levels. It was revealed that YAP/TAZ target genes were declined in T98G- and U251-sh-S100A16 cells (**Fig. 7B**), and raised in U87- and A172-S100A16 cells (**Fig. 8B**). To observe whether S100A16 affects YAP/TAZ activity by boosting its nuclear localization, YAP/TAZ protein levels in cytoplasmic and nuclear components were separately measured. In U251-shS100A16 cells (**Fig. 7C**), YAP protein was significantly reduced in nucleus compared to controls, but increased in U87-S100A16 nucleus (**Fig. 8C**). In addition, IF showed sh-S100A16 increased YAP expression and accumulation in the cytoplasm of U251 cells, as well as the mobility of YAP from the nucleus to the cytoplasm (**Fig. 7D**). Conversely, nuclear localization of YAP was enhanced in U87-S100A16 cells (**Fig. 8D**). In total, these findings suggest that S100A16 inhibited Hippo signaling pathway, thus regulating YAP behavior and cellular distribution in glioma cells.

### LATS1 mediated S100A16-promoted tumorigenesis

**Figure 9:**
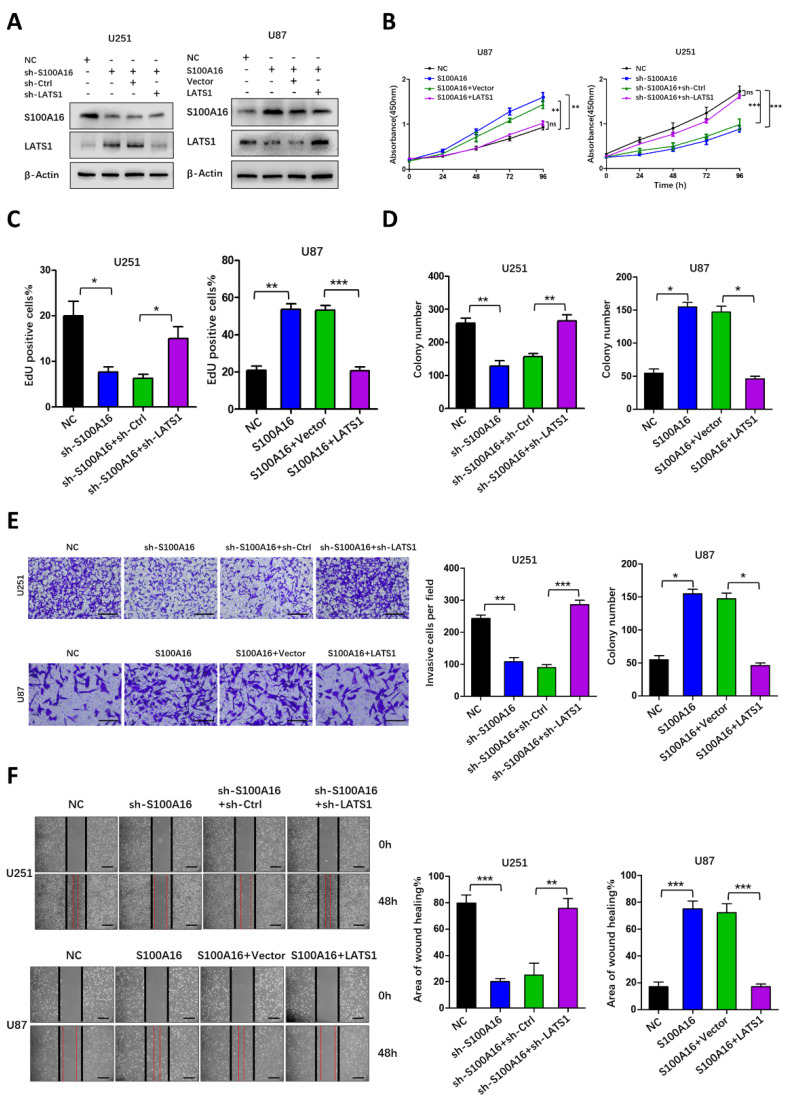
** LATS1 mediated S100A16-dependent proliferation, migration and invasion of glioma cells**. **(A)** Western Blot assay of lysates generated from U251 cells transfected with shS100A16 and shLATS1, or U87 cells transfected with S100A16 and LATS1 plasmids. **(B)** CCK-8 assay of cell viability of the modified U251 and U87 cells; **(C)** EdU assay of proliferative capacity of the modified U251 and U87 cells; **(D)** Plate colony of proliferative ability of both modified cells. **(E)** Transwell assay of invasive ability of both modified cells;** (F)** Wound healing test of migration ability of both modified cells.

We have identified that S100A16 induced a cascade response in the Hippo pathway. Among them, the upstream MST1/2 protein and its phosphorylation were not altered obviously, while LATS1 protein was significantly different. Despite changes in its phosphorylation, it may be due to obvious changes in total LATS1 protein. These allowed us to consider that the effects of S100A16 on tumorigenesis were dependent on LATS1 protein. Therefore, we co-transfected shRNA targeting LATS1 into U251-shS100A16, and co-transfected LATS1 cDNA into U87-S100A16. Overexpression or knockdown efficiency was assessed by western blot. As shown in **Fig. 9A**, S100A16 expression was not influenced by LATS1, whereas knockdown of LATS1 in U251-shS100A16 cells reduced the upregulated LATS1 expression due to S100A16 knockdown, ectopic expression of LATS1 in U87-S100A16 cells restored the poor LATS1 expression due to S100A16 overexpression. We also conducted a series of functional experiments to demonstrate the effect of LATS1 in S100A16-independent proliferation, migration and invasion. CCK-8 (**Fig. 9B**) showed that down-regulation of LATS1 restored the proliferation rate of U251 cells inhibited by shS100A16, while upregulation of LATS1 reduced the growth ability of U87 cells transfected by S100A16. This was consistent with the results of EdU (**Fig. 9C, Fig. S5A**) and colony-forming assays (**Fig. 9D, Fig. S5B**). Moreover, transwell (**Fig. 9E**) and wound healing assays (**Fig. 9F**) suggested that sh-LATS1 reversed the inhibitory impact of sh-S100A16 on glioma migration and invasion in U251 cells. In contrast, over-expression of LATS1 in U87-S100A16 cells obtained the inverse effects. These data support that LATS1 could be a potential downstream and active target of S100A16 in the development of glioma.

### S100A16 promotes LATS1 ubiquitination for proteasomal degradation

**Figure 10:**
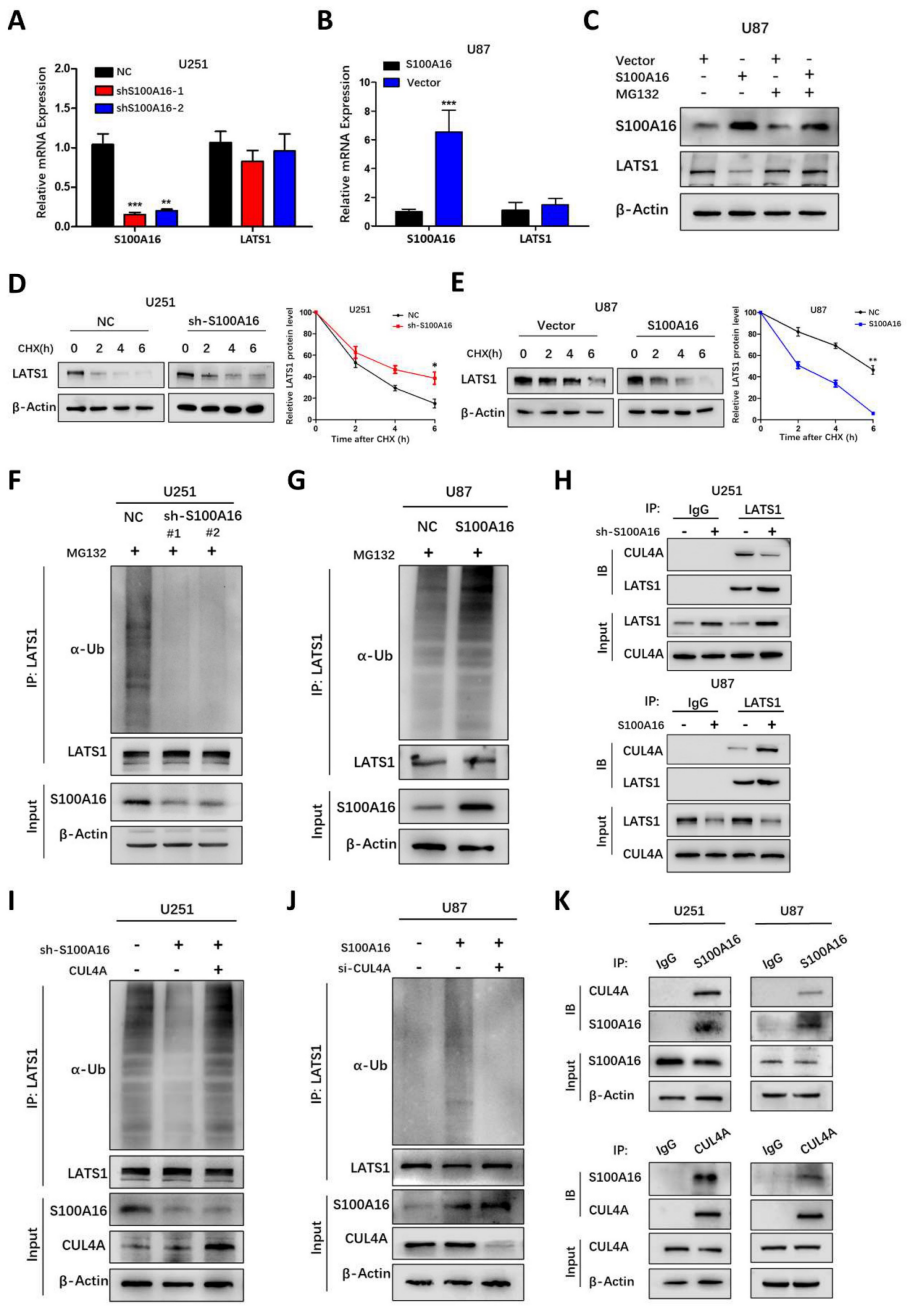
**S100A16 destabilized LATS1 by CUL4A-mediated ubiquitination. (A-B)** qRT-PCR of S100A16 and LATS1 mRNA levels in U251-shS100A16 and U87-S100A16 cells. **(C)** Western blot of LATS1 protein in transduced U87 cells after 10μM MG132 treatment for 8 hours. **(D-E)** Protein degradation of LATS1 in U251-shS100A16 and U87-S100A16 cells treated with 50ug/ml CHX at indicated time points. **(F-G)** Ubiquitination assays of LATS1 in U251-shS100A16 and U87-S100A16 cells after 10μM MG132 treatment for 8 hours. **(H)** IP detection of interaction between CUL4A and LATS1 in U251 and U87 cells. **(I-J)** Ubiquitination assays of LATS1 in U251 cells co-transfected sh-S100A16 and CUL4A, or U87 cells co-transfected sh-S100A16 and CUL4A after 10μM MG132 treatment for 8 hours. (K) IP analysis of the interaction between S100A16 and CUL4A in U251 and U87 cells.

To elucidate the mechanism by which S100A16 controls LATS1 activity, we tested whether S100A16 affects LATS1 transcriptionally. Interestingly, alteration of S100A16 had no obvious effect on mRNA levels of LATS1 (**Fig. 10A, B**). This led to the possibility that S100A16 modulates LATS1 protein stability. We next determined LATS1 protein levels in GBM cells pretreated with the proteasome inhibitor MG132. As shown in **Fig. 10C**, MG132 restored LATS1 levels after overexpressing S100A16 in U87 cells. In addition, a protein synthesis inhibitor, CHX, was added to GBM cells to detect the half-life of LATS1 protein. The result showed that LATS1 half-life was lengthened in U251-shS100A16 cells (**Fig. 10D**), but was lessened in U87-S100A16 cells (**Fig. 10E**). Thus, S100A16 could promote proteasome-mediated degradation of LATS1.

Ubiquitin (Ub) is a classical post-translational modification involved in proteasomal degradation. Thus, we investigated whether S100A16 regulates LATS1 proteolysis through the ubiquitin pathway in GBM cells pre-treated with MG132. The results suggested that endogenous LATS1 ubiquitination was decreased in U251-shS100A16 cells (**Fig. 10I**), while increased in U87-S100A16 cells (**Fig. 10J**). In total, the data above indicated that S100A16 mediated the stability of LATS1 in a ubiquitin-independent way.

### S100A16 destabilizes LATS1 via E3 ligase CUL4A

Ubiquitination regulation is usually catalyzed by *E3* ubiquitin ligase. CUL4A^DCAF^, as a ubiquitin E3 ligase complex, was reported to modulate LATS1 ubiquitination and protein stability at the post-translational level. This implies that S100A16 may have a potential role in promoting CUL4A^DCAF^-mediated ubiquitination of LATS1 in glioma. Firstly, we performed IP experiments pulling down complexes with LATS1 antibody and the results indicated that LATS1 combined with CUL4A, DCAF1 and DDB1 (**Fig. S6A**). We further found that the decreased expression of LATS1 by S100A16 could be partially recovered under the inhibition of CUL4A, DCAF1 and DDB1, but the effect was more marked by CUL4A (**Fig. S6B-D**). CUL4A is the core component of this E3 ligase, and a recent study has shown that the recruitment of CUL4A could directly induce LATS1 ubiquitination. Therefore, we focus on the link among CUL4A, LATS1 and S100A16. As shown in** Fig. 10H,** S100A16 knockdown decreased CUL4A bounding to LATS1, whereas overexpression of S100A16 increased CUL4A bounding to LATS1. Thus, S100A16 significantly influenced the amount of CUL4A complexed with LATS1. To further confirm that S100A16 regulates LATS1 protein levels through CUL4A-mediated ubiquitination, we transfected CUL4A siRNA or cDNA into glioma cells pre-treated with MG132. In the presence of U251-shS100A16 cells (**Fig. 10I**), up-regulating CUL4A reversed the reduced LATS1 ubiquitination by sh-S100A16. In the U87-S100A16 cells (**Fig. 10J**), silencing CUL4A attenuated the increased LATS1 ubiquitination caused by S100A16 overexpression. We also performed CO-IP experiments to confirm the existence of the interaction of S100A16 and CUL4A (**Figure 10K**). Overall, these results support our hypothesis that S100A16 destabilizes LATS1 by facilitating CUL4A-mediated ubiquitination for proteasomal degradation.

### S100A16 promotes tumor growth *in vivo*

**Figure 11:**
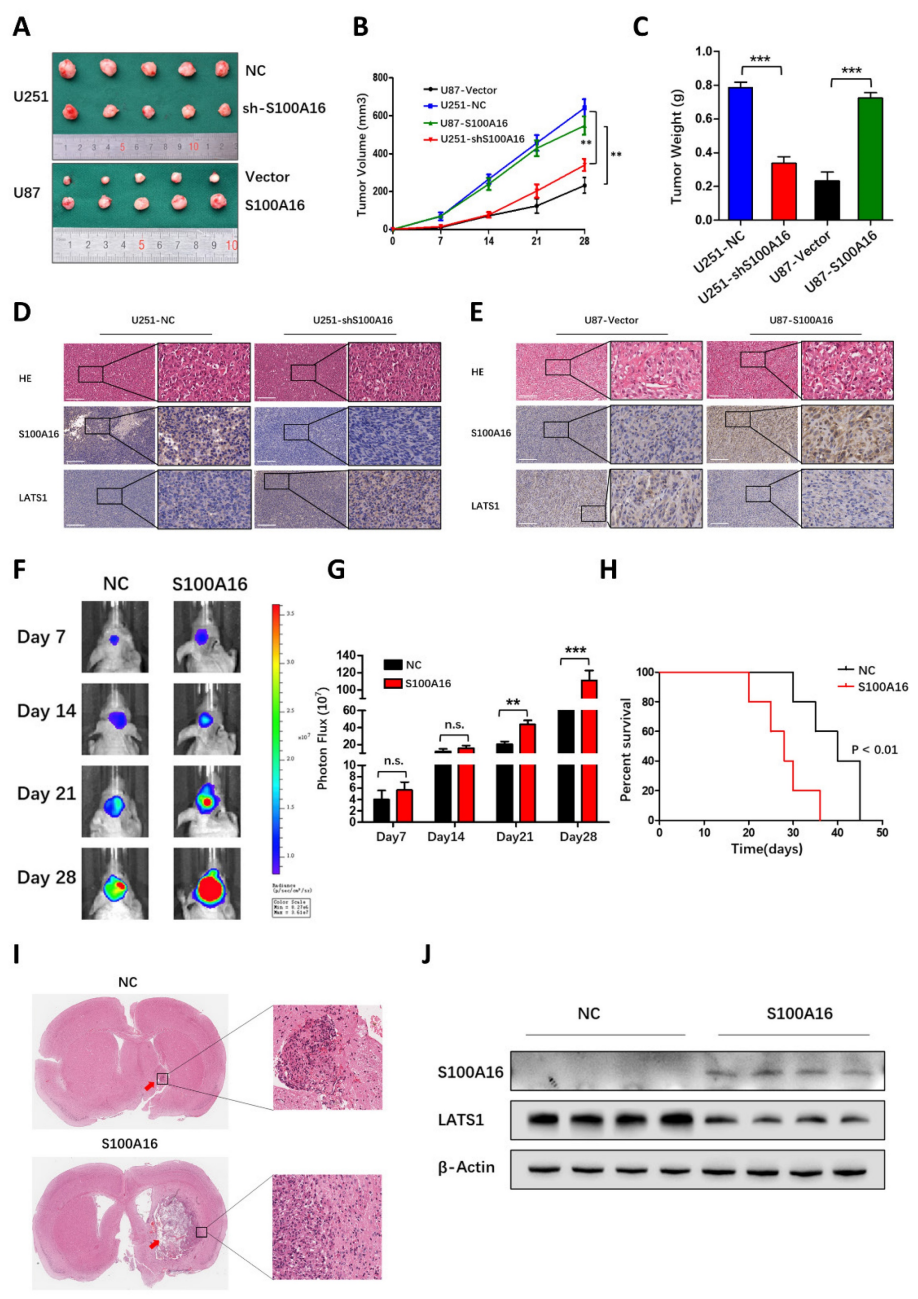
** S100A16 promoted glioma growth *in vivo*. (A)** Subcutaneous xenografts from mice implanted with U251-NC, -shS100A16 cells and U87-vector, -S100A16 cells; **(B)** Growth curves of subcutaneous tumor from indicated mice; **(C)** Subcutaneous tumor weight from indicated mice. **(D-E)** IHC staining of S100A16 and LATS1 in subcutaneous tumor specimens from infected U251 and U87 cells. **(F)** Bioluminescence images of orthotopic tumors from nude mice bearing U87-NC and -S100A16 cells; **(G)** Quantification analysis of orthotopic tumor volume. **(H)** K-M survival curve of nude mice from U87-NC or U87-S100A16 transplanted intracranial xenografts; **(I)** Representative HE staining of orthotopic xenografts from indicated groups. **(J)** Western blot analysis of S100A16 and LATS1 from transplanted orthotopic tumors.

**Figure 12:**
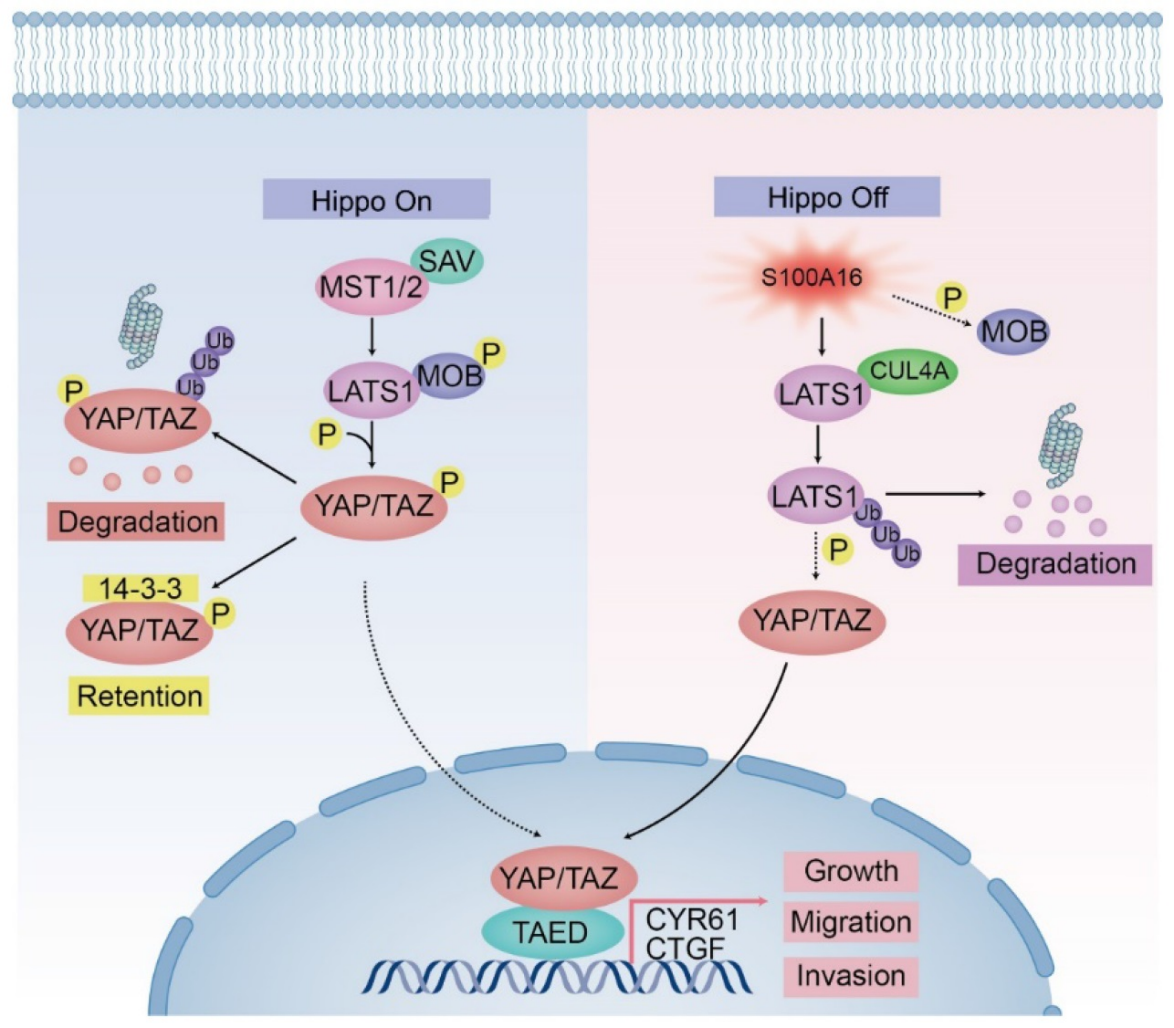
** A model of the regulator mechanism of S100A16 in Glioma.** Left half represents the Hippo signaling cascades in state “on” that block YAP entry to the nucleus. Right half indicates that S100A16 lead to Hippo signaling pathway in state “Off”, showing the inactivation of MOB phosphorylation, the promotion of CULA4-mediated LATS1 ubiquitination for proteasomal degradation, which results in YAP activation and glioma tumorigenesis.

To prove the promoting effect of S100A16 *in vivo*, we infected glioma cells with S100A16 shRNA or cDNA lentivirus, and constructed the subcutaneous or orthotopic animal model. Representative images of subcutaneous tumors were shown in **Fig. 11A**. The oncogenic efficiency of S100A16 was confirmed and evaluated by tumor volume (**Fig. 11B**) and tumor weight (**Fig. 11C**). Compared with controls, tumor growth was decreased in U251-shS100A16 cells, whereas increased in U87-S100A16 cells. IHC showed that S100A16 was inversely linked with LATS1 expression, indicating possible interaction of these two proteins (**Fig. 11D**). We have previously validated the LATS1 antibody specificity using LATS1 KD glioma cells (**Fig. S7**). In an orthotopic xenograft model, the tumor growth was monitored by bioluminescence signals, and representative images were shown in **Fig. 11E**. Quantitative analysis demonstrated that S100A16 significantly enlarged the tumor volume *in vivo* (**Fig. 11G**). In survival curve, overexpression of S100A16 significantly shortened the survival time of nude mice bearing orthotopic tumors (**Fig. 11H**). The HE-stained orthotopic tumors are presented in **Fig. 11I**, with an expanded tumor infiltration in the S100A16-transfected group. To confirm the relationship between S100A16 and LATS1, we chose four pairs of resected tumors at random from shCtrl- and S100A16-transfected groups, respectively. Western blot showed that overexpression of S100A16 lead to a reduction of LATS1 expression (**Fig. 11J**). These results are consistent with the previously proposed mechanism that S100A16 could down-regulate the downstream protein LATS1, and play an oncogenic role in glioma. A schematic diagram of S100A16 promoted glioma progression was concluded in **Fig. 12**.

## Discussion

Glioma is the most frequent and lethal type of primary CNS malignancy, with a high surgical risk, easy recurrence after surgery and poor survival prognosis 21. Hence, it is essential to understand the mechanism that drives glioma progression, and thus identify novel target genes and explore promising therapeutic approaches. S100 family, containing about 25 members, has been identified to be implicated in diverse biological processes of cancer. In our previous study, we developed an S100 family-based prognostic signature consisting of five genes of S100A11, S100A16, S100B, S100PBP and S100A13 for glioma patients 22. Among them, S100A16 is a new S100 family member, with a more stable and conservative structure than others. However, S100A16's molecular mechanism and function in human glioma have not been well understood.

In this study, we first found that S100A16 expression differed between normal brain and glioma tissues, which varied across tumor types, based on GEPIA database. This indicated that S100A16 possesses a dual role, acting as a tumor suppressor or tumor promoter in different malignancies. In human brain tissues, S100A16 is highly expressed in glioma relative to non-neoplastic brain tissues, which was also verified in the TCGA, CGGA and GEO databases. In addition, S100A16 was significantly associated with glioma patients' prognosis, meaning that the survival time of patients with high-S100A16 expression was greatly shorter than those with low S100A16. To further confirm the role of S100A16, we determined S100A16 expression in real clinical specimens, and conducted functional experiments *in vitro* and vivo. It was first established that S100A16 upregulation greatly enhanced glioma proliferation, migration and invasion. These suggested that S100A16 might act as an oncogene promoter in glioma.

The potential mechanisms of S100A16-mediated glioma development require deep exploration. Our bioinformatic analysis implied that the Hippo signaling pathway, MTOR pathway, WNT pathway et.al might be implicated in glioma malignant progression regulated by S100A16. Previous studies have reported that MTOR and WNT signaling play important roles in cellular growth, migration and survival in glioma progression 23-25. We also found that S100A16 could be a key regulator of WNT pathway in acute renal injury 26, but their association in glioma has not been reported. In the present study, pathway analysis revealed that the Hippo signaling pathway is the most significantly enriched in glioma. The correlation analysis also showed an obvious positive correlation between S100A16 and YAP, as well as its target genes. These suggested that S100A16 may be highly related to Hippo pathway in glioma. Hippo pathway is a novel and highly conserved pathway, which is responsible for organ size control, tissue regeneration, and cancer suppression 27. Recent studies have suggested that the Hippo pathway could be influenced by intrinsic and extrinsic factors, which is critical in CNS maturation and brain tumor formation 20,28. Hence, we investigated the alterations of various signaling molecules in this pathway by S100A16. We found that S100A16 did not affect MST1/2 expression, but decreased LATS1 protein and MOB phosphorylation, which allowed YAP to enter the nucleus triggering the downstream targets CYR61 and CTGF, which are known to stimulate the angiogenesis and tumor growth 29,30. These findings indicated that S100A16 regulated the Hippo signaling pathway, and the cascade response may be ascribed to the inactivation of LATS1 by S100A16, resulting in this pathway being in an “off” state.

LATS1 encodes a serine/threonine kinase that negatively regulates YAP, and exhibits anti-tumor activity by restricting cell proliferation or promoting apoptosis 31. LATS1 is susceptible to being a molecular target that is mediated by various factors,and is consequently engaged in the development of glioma 32-34. In our study, S100A16 was shown to have a promotive impact on glioma proliferation, invasion and metastasis, which might be achieved by knockdown of LATS1. Importantly, our data showed that S100A16 did not change mRNA levels of LATS1, giving a hint that S100A16 down-regulated LATS1 post-translationally but not transcriptionally. Ubiquitination is a common post-translational modification that marks proteasomes for degradation and regulates diverse signaling pathways 35. The specific recognition of ubiquitination targets is dependent on E3 ubiquitin ligase. Li, et al. demonstrated that CRL4-DCAF1 inhibits the output of the Hippo signaling pathway by promoting ubiquitination of LATS1^36^,^37^. CUL4A is the core component of the CRL4-DCAF1 E3 ubiquitin ligase complex. Another recent study revealed that the recruitment of CUL4A ubiquitin ligase directly induced LATS1 ubiquitination and degradation 38. In the present study, we demonstrated that S100A16 could promote the ubiquitination of LATS1 protein by enhancing its binding to CUL4A. It revealed a new mechanism by which S100A16 promoted the development and progression of glioma.

However, our study still has several limitations. The specific mechanism of interaction among S100A16, CUL4A and LATS1, as well as the binding sites warrants more investigation in our future work. Moreover, there are more than one E3 ubiquitin ligase for LATS1, further investigations are needed to characterize other CUL4A adaptors or additional E3 ligases in the regulation of tumor progression by S100A16.

In conclusion, we discovered that S100A16 was highly expressed in glioma and related to prognostic survival. Furthermore, S100A16 acted as a negative regulator of the Hippo signaling pathway to assist in glioma proliferation, invasion and migration, the mechanism by which was probably to interact with and promote CUL4A, an E3 ubiquitin ligase binding to LATS1 and induce its ubiquitination degradation, which resulted in YAP/TAZ entry into the nucleus, targeting the downstream genes CTGF and CYR61. Consequently, these data revealed a new insight into glioma pathogenesis and identified S100A16 as a prospective molecular target for glioma patients' diagnosis, treatment and prognosis.

## Supplementary Material

Supplementary figures and tables.
