# Supplementary material for: S100 Calcium Binding Protein A16 Promotes Cell Proliferation by triggering LATS1 ubiquitin degradation mediated by CUL4A ligase to inhibit Hippo pathway in Glioma development
Source: Int J Biol Sci. 2023 Apr 2;19(7):2034–52. doi: 10.7150/ijbs.79924 (PMC10158029; doi:10.7150/ijbs.79924)
Supplement: Supplementary file 1 — Supplementary figures and tables. [file ijbsv19p2034s1.pdf]

Supplemental information

Table S1. Plasmid Sequences were available in this study.

shRNAs or siRNAs	Sequences (5′-3′)
sh-S100A16-1	CAGAACCTGGATGCCAATCAT
sh-S100A16-2	CAGTCATTGTCCTGGTGGAAA
sh-NC	TTCTCCGAACGTGTCACGT
sh-LATS1	GATTACAACTTCACCTATTACCTCG
	AGGTAATAGGTAGAAGTTGTAATC
sh-NC	CCTAAGGTTAAGTCGCCCTCGCT
	CGAGCGAGGGCGACTTAACCTTAGG
si-NC	GGCUCUAGAAAAGCCUAUGC
siCUL4A	GCATGTGGATTCAAAGTTA
siDCAF1	CGAGAAACTGAGTCAAATGAAC
siDDB1	CCAUCGAUGAGAUCCAGAA

Table S2. Primer Sequences were available in this study.

	Forward primer	Reverse primer
S100A16	AAGGCTGCGGATAAGCTCATC	CCGCCTATCAAGGTCCAGT
CTGF	TGGAAGAGAACATTAAGAAGGGCA	TGCAGCCAGAAAGCTCAAAC
Cyr61	GCAAGGAGCTGGGATTCGAT	ATTCCAAAAACAGGGAGCCG
LATS1	AATTTGGGACGCATCATAAAGCC	TCGTCGAGGATCTTGGTAACTC
β-actin	AGTGTGACGTTGACATCCGTA	GCCAGAGCAGTAATCTCCTTCT

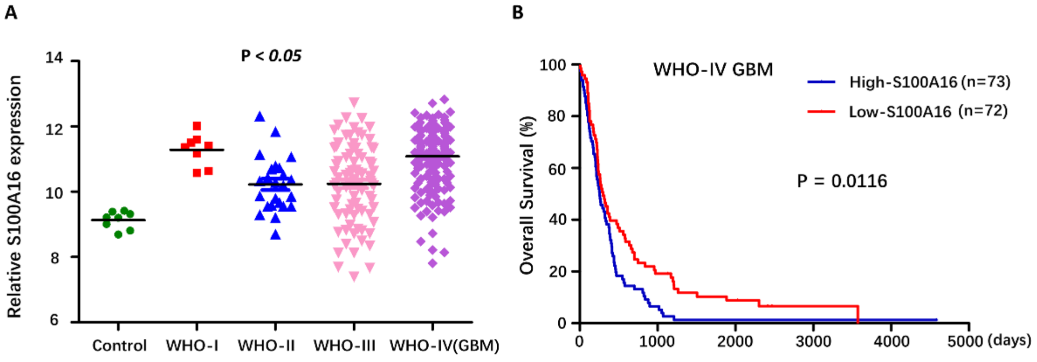

Fig. S1: S100A16 expression and survival analysis based on GEO database. (A) S100A16 mRNA expression levels in normal controls and different grades of glioma tissues; **(B)** Survival analyses of GBM patients with high- and low-S100A16 expression.

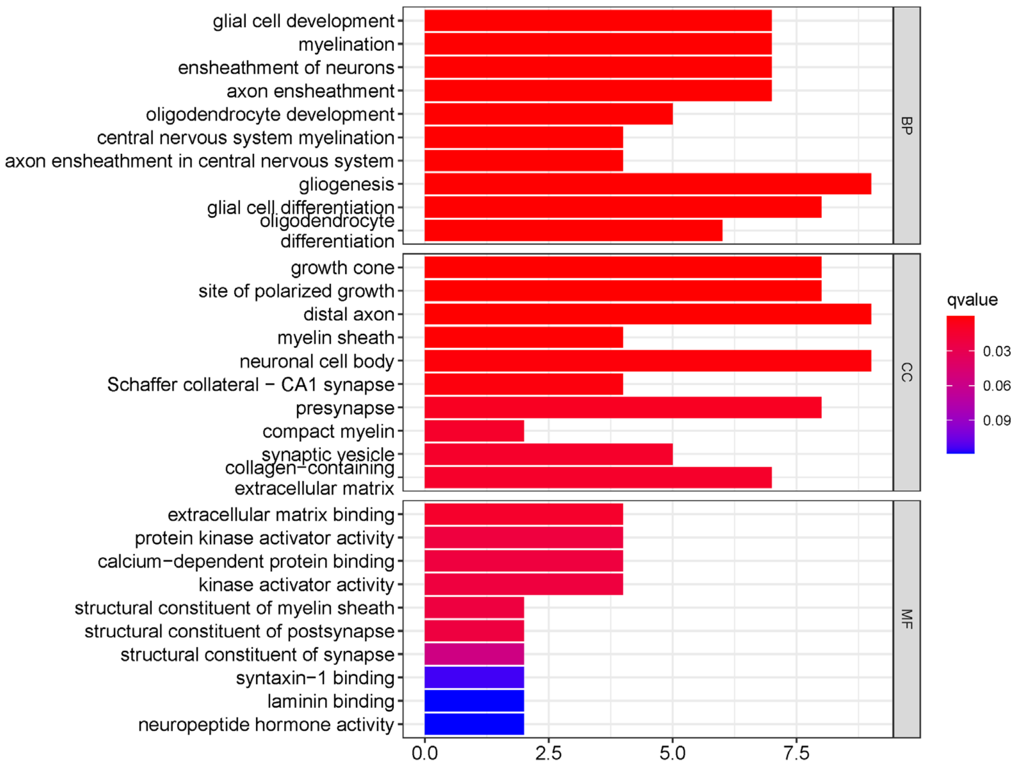

Fig. S2: GO analyses of DEGs between high- and low-S100A16 groups in glioma, including biological process, cell components, and molecular function.

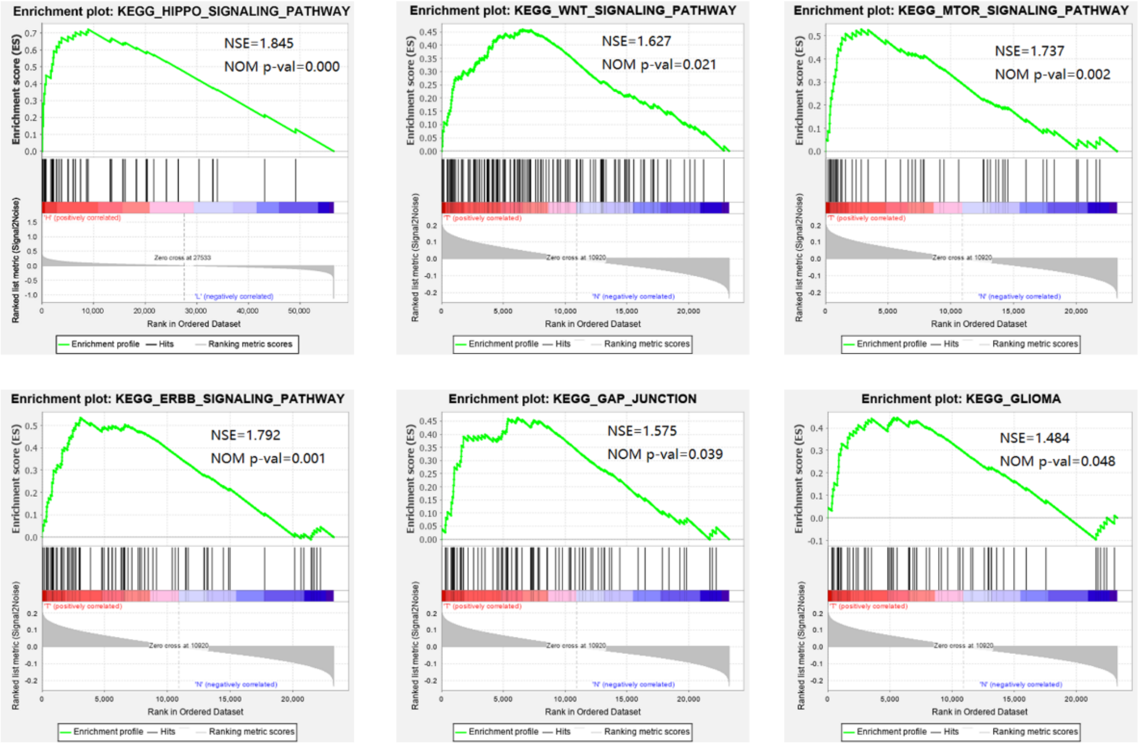

Fig. S3: Gene set enrichment analysis identifying S100A16-related pathways in Glioma.

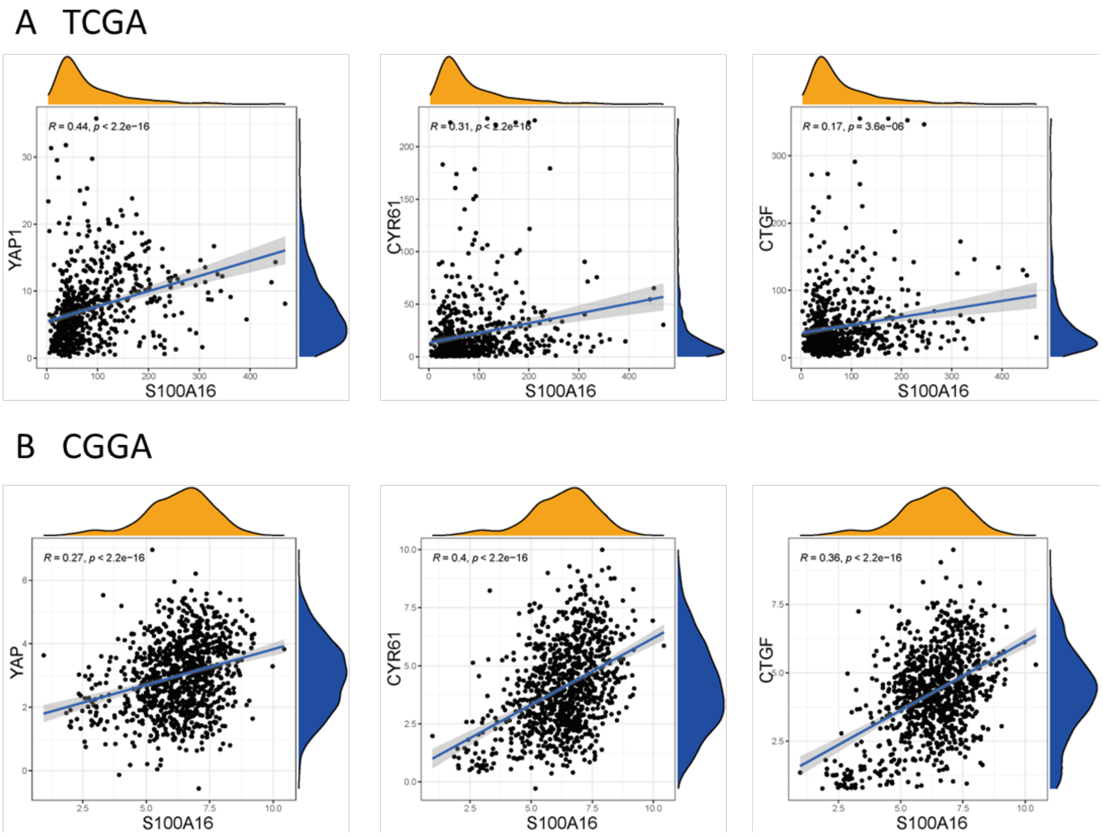

Fig. S4: Association of S100A16 expression with YAP, CTGF and CYR61 in human glioma samples based on TCGA and CGGA databases.

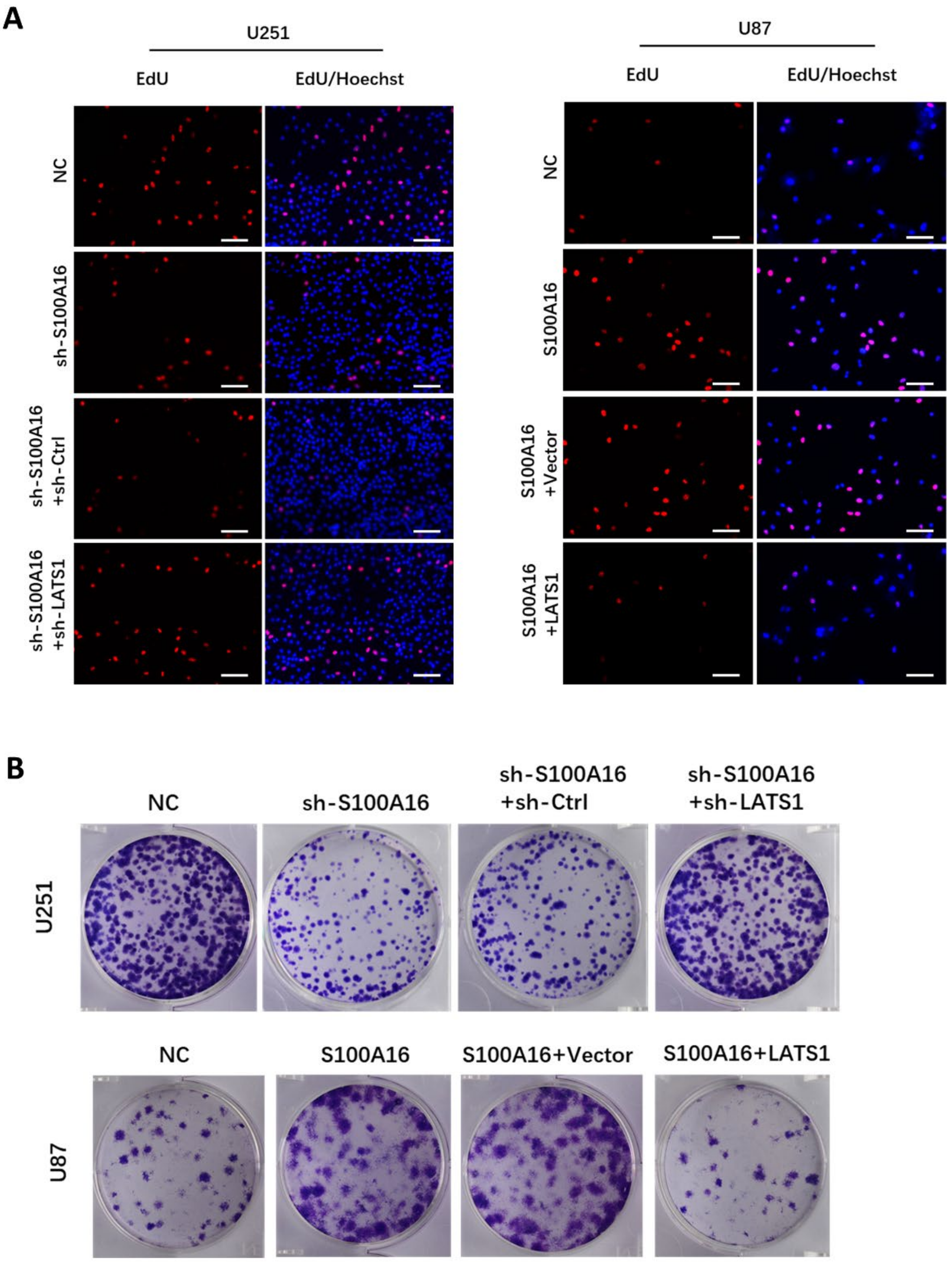

Fig. S5: (A) EdU images of U251 cells transfected with shS100A16 and shLATS1, or U87 cells transfected with S100A16 and LATS1 plasmids; **(B)** Representative images of plate formation colonies of the indicated cells.

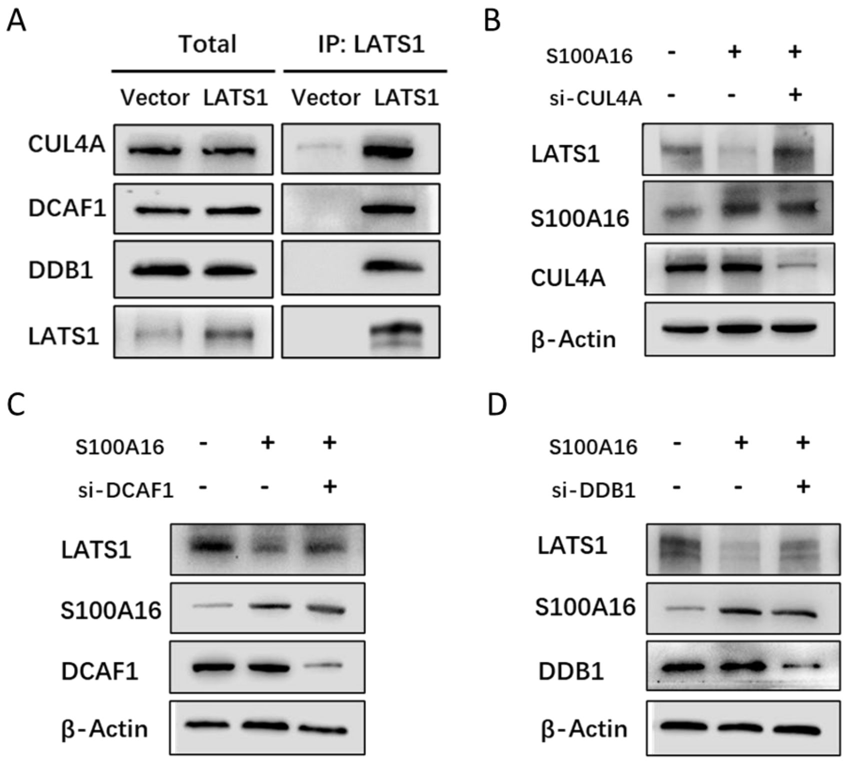

Fig. S6: LATS1 Associates CUL4A^{DCAF1} ubiquitin ligase complex. (A) IP experiments with anti-LATS1 were subjected to immunoblotting with indicated antibodies in U87 cells. **(B-D)** Altered expression of LATS1 protein by S100A16 in U87 cells transfected with si-CUL4A, si-DCAF1, si-DDB1.

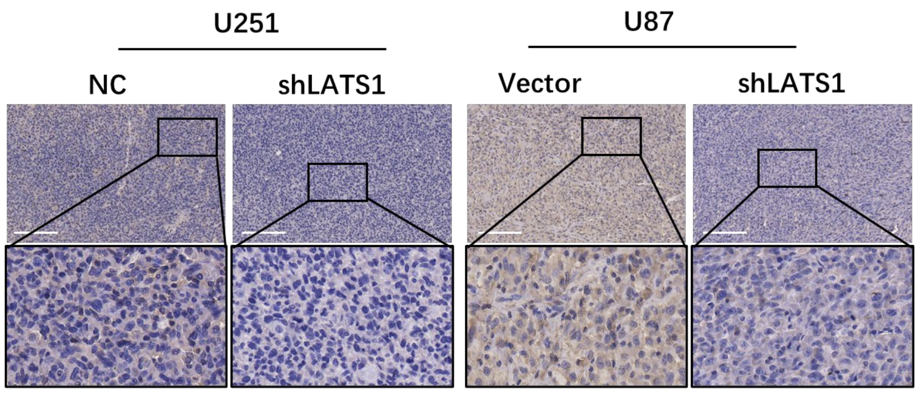

Fig. S7: IHC staining of LATS1 in subcutaneous tumors using glioma cells transfected with shLATS1.
